# Total cost estimation for implementing genome-enabled selection in a multi-level swine production system

**DOI:** 10.1186/1297-9686-46-32

**Published:** 2014-05-19

**Authors:** Caitlyn E Abell, Jack CM Dekkers, Max F Rothschild, John W Mabry, Kenneth J Stalder

**Affiliations:** 1 Iowa State University, 109 Kildee Hall, Ames, IA 50011, USA

## Abstract

**Background:**

Determining an animal’s genetic merit using genomic information can improve estimated breeding value (EBV) accuracy; however, the magnitude of the accuracy improvement must be large enough to recover the costs associated with implementing genome-enabled selection. One way to reduce costs is to genotype nucleus herd selection candidates using a low-density chip and to use high-density chip genotyping for animals that are used as parents in the nucleus breeding herd. The objective of this study was to develop a tool to estimate the cost structure associated with incorporating genome-enabled selection into multi-level commercial breeding programs.

**Results:**

For the purpose of this deterministic study, it was assumed that a commercial pig is created from a terminal line sire and a dam that is a cross between two maternal lines. It was also assumed that all male and female selection candidates from the 1000 sow maternal line nucleus herds were genotyped at low density and all animals used for breeding at high density. With the assumptions used in this analysis, it was estimated that genome-enabled selection costs for a maternal line would be approximately US$0.082 per weaned pig in the commercial production system. A total of US$0.164 per weaned pig is needed to incorporate genome-enabled selection into the two maternal lines. Similarly, for a 600 sow terminal line nucleus herd and genotyping only male selection candidates with the low-density panel, the cost per weaned pig in the commercial herd was estimated to be US$0.044. This means that US$0.21 per weaned pig produced at the commercial level and sired by boars obtained from the nucleus herd breeding program needs to be added to the genetic merit value in order to break even on the additional cost required when genome-enabled selection is used in both maternal lines and the terminal line.

**Conclusions:**

By modifying the input values, such as herd size and genotyping strategy, a flexible spreadsheet tool developed from this work can be used to estimate the additional costs associated with genome-enabled selection. This tool will aid breeders in estimating the economic viability of incorporating genome-enabled selection into their specific breeding program.

## Background

The common method to estimate breeding values and rank animals based on genetic merit is known as traditional BLUP (best linear unbiased prediction) selection. Traditional BLUP selection relies on phenotypic information measured directly on selection candidates and their relatives to predict the genetic merit for all animals. If molecular information is included in the selection program, historically it would have been in the form of marker-assisted selection for which only a few markers are used [1].

With advances in molecular technology and new biological tools, researchers have established ways to incorporate variation at the DNA level into breeding programs. When breeders incorporate the variation from genomic information into a selection strategy, it is known as genome-enabled selection. This information is used to enhance traditional breeding value estimation. Estimating an animal’s genetic merit at the molecular level may improve estimated breeding value (EBV) accuracy [2]. This improved accuracy could further increase the rate of genetic gain for the population. Traits that are lowly heritable, difficult to measure, sex-limited, measured later in life, or measured after slaughter, have the greatest potential for accuracy improvement when using genome-enabled breeding value estimation compared to traits that can be directly measured on all the candidates before selection.

Using a low-density marker panel can be an effective method to reduce genotyping costs once the initial dataset has been established. An initial population is needed to determine population haplotypes so that imputation can be used to infer genotypes from a low-density marker panel. Inferring high-density genotypes from a low-density panel is known as imputation [3]. When imputation is used, selection candidates are genotyped using low-density panels and the actual selected animals are commonly re-genotyped using a high-density marker panel. To further reduce costs, companies may choose to genotype only males. While this saves costs, genomic EBV accuracy for genotyped animals may be reduced, especially for sex-limited or novel traits for which phenotypic data are not routinely collected.

The objective of this study was to develop a tool to estimate the cost structure associated with incorporating genome-enabled selection into commercial breeding programs. Estimating an animal’s genetic merit using genomic information can improve the accuracy of EBV; however, this improved accuracy should be large enough to recover the costs associated with implementing genome-enabled selection.

## Methods

Most commercial market hogs are the offspring from a mating between a female that is a cross between two maternal lines and a male that is from a terminal line. The three lines can be purebred, synthetic, or some composite of the same type. Therefore, each maternal line contributes 25% of the genetic material to commercial animals, and the terminal line makes up the other 50%. One maternal line is typically derived from a Landrace population, while the other maternal line is usually derived from a Large White population. The terminal line is often derived from a Duroc population. Each maternal line nucleus was assumed to have 1000 sows while the terminal nucleus had 600 sows. All three lines must be selected for improved performance at the commercial level, and thus, EBV must be estimated for each line. The terminal line is typically selected based on a terminal sire index that can consist of growth, meat quality, and carcass traits. The maternal lines are often selected based on a maternal line index, which comprises reproductive traits that are more heavily emphasized in the index and some terminal market traits.

### Initial costs

When considering the costs associated with genome-enabled selection, one has to consider not only the genotyping costs, but also other ancillary expenses. In the present analysis, genotyping costs were assumed to be US$115 and US$55 for the high- and low-density panels, respectively. These costs include the cost of the genotyping and all other costs associated with sample collection, DNA isolation and storage, shipping, etc. Genotyping costs will vary depending on the number of animals genotyped and the company used. Additionally, genotyping costs will change (increase or decrease) as new tests become available. For each of the three lines, it was assumed that the initial data consisted of 2000 ancestor animals that were genotyped using the high-density marker panel.

Developing genome-enabled EBV requires more time and computing power compared to traditional BLUP EBV. For this analysis, it was assumed that eight months were required to analyze the initial dataset and develop the program that will be implemented for genetic evaluations. Based on personal communications with a large swine genetics company, an additional 8 hours was assumed to be needed with each weekly evaluation to prepare data and to ensure the program runs without errors. It was assumed that the EBV development and additional weekly work would cost US$60/hour in employee wages and benefits.

A US$50 000 investment was assumed for computing infrastructure. This cost includes equipment and labor associated with set-up. For this study, the infrastructure cost was distributed across the three lines included in the breeding program based on the relative proportion of nucleus sows in each line. For this analysis, it was assumed that the costs of the investment are expected to be recovered within three years. In other words, a three-year planning horizon was assumed. The infrastructure costs were assumed to have no salvage value at the end of the planning horizon. A 5% discounting rate [4] was used to calculate the present value of the nominal annual costs associated with genome-enabled selection.

When assessing cost structure within a genome-enabled selection program, the infrastructure costs are nominal relative to the annual genotyping costs. Therefore, a longer planning horizon would not greatly impact the total annual costs associated with incorporating genome-enabled selection into the breeding program. A longer planning horizon would allow accumulated genetic improvement in multiple generations to help offset the additional costs.

### Annual costs

Once an initial dataset has been collected and analyzed, routine genotype collection can be scheduled for selection candidates within each line in the breeding program. All the offspring produced from the nucleus breeding herd are potential selection candidates. The number of potential selection candidates and the genetic sampling will determine how many selection candidates in a given year must be genotyped. For this analysis, all male and female selection candidates were expected to be genotyped at low density and then all animals used for actual breeding were re-genotyped at high density in both maternal lines. While genotyping both males and females is optimal for increased annual genetic gain and reduced inbreeding, it is not required to obtain genetic improvement using genome-enabled selection [5]. Only male selection candidates were genotyped for the terminal line, and boars selected to become sires within the nucleus were re-genotyped at high density. Only males are genotyped in the terminal line since most terminal line selection indices do not include sex-limited traits, reducing the value of genotyping females. This genotyping scheme is representative of the strategy currently implemented in the swine industry. Another way to reduce the annual genotyping costs would be to genotype only a proportion of available male and/or female selection candidates. Henryon et al. [6] showed that only 5 to 20% of the selection candidates must be genotyped in order to capture most of the genetic gain realized from genotyping all of the selection candidates. The animals to be genotyped could be preselected based on their genetic potential estimated using traditional BLUP methods [7].

### Nucleus production

Genetic suppliers have different marketing schemes. Revenues are obtained by selling boars, selling semen doses, or charging weaned pig fees. However, a weaned pig fee can be converted into the price of a dose or boar based on how many commercial weaned pigs are produced by nucleus boars. For the maternal lines, it was assumed that, on average, each litter in the nucleus herd consisted of 11 pigs born alive [8] with a 1:1 sex ratio [9]. Each sow was assumed to have 2.3 litters a year, on average [8]. The combined pre-weaning and nursery mortality was assumed to be 12% [8]. These assumptions result in 22 264 animals (1000 sows × 2.3 litters × 11 pigs × 0.88 survival; 11 132 males and 11 132 females) to be genotyped annually at low density in each maternal line. With 5% and 20% selection for males and females [10], respectively, 557 males (11 132 × 0.05) and 2226 females (11 132 × 0.20) will be re-genotyped with the high-density panel annually in each maternal line. These represent the number of animals selected, but not all animals will be successful, reproductive members of the breeding herd.

The average sow production in the terminal line nucleus herd was assumed to be 10 pigs born alive per litter with a 1:1 sex ratio [9] and 2.1 litters per sow per year [8]. The total mortality through the nursery was assumed to be 12%, as for the maternal lines. This means that 5544 male pigs are produced and genotyped at low density in the terminal line nucleus herd, annually. Assuming 5% male selection, 277 males would be re-genotyped with a high-density panel.

The top 5% of the boars produced in the nucleus herd based on genome-enabled EBV are assumed to be used as nucleus replacements in the maternal and terminal lines. The top 60% of male candidates in the sire line were used in the commercial production system for the terminal line. Boars were assumed to produce 25 semen doses weekly with 15% of doses being discarded due to semen quality issues [11]. It was assumed that 25% of selected boars would not reach production due to infertility, disease, etc. All boars used for reproduction within the nucleus or at the commercial level were assumed to be used to maximum capacity, meaning that all doses that are not discarded are used for insemination. This means that 461 282 and 2 756 754 doses were produced annually from the maternal line boars and terminal line boars, respectively. For example, the annual doses produced in a terminal line would be calculated as 5544 selection candidates × 75% productive × 60% used × 25 doses per week × 85% viable semen × 52 weeks. This formula assumes that the boars have an average production life of one year.

The total number of weaned pigs produced each year from the semen doses collected was calculated as 1 940 845 and 10 544 584 for the maternal and terminal lines, respectively. The number of total weaned pigs produced each year was calculated by multiplying the expected number of litters by the number of pigs weaned in a litter. This was based on 10% pre-weaning mortality in the commercial herd, two doses of semen per sow serviced, and an 85% farrowing rate for commercial sows with 10 and 11 pigs born alive per litter in the terminal and maternal lines, respectively [8]. For example, the number of weaned pigs produced from the terminal boars was calculated as 2 756 754 doses / 2 doses per service × 85% farrowing rate × 10 pigs/litter × 90% survival to weaning. For the maternal lines, 40% of the gilts developed were assumed to never produce a litter [12], and for the sows that farrowed at least one litter, it was assumed that each sow produced 35 weaned pigs per lifetime, on average [8]. For this study, it was assumed that the commercial producers would have an internal multiplication program for replacement gilts, and thus, genetic improvement at the commercial level would be realized through purchasing semen doses and/or boars from the genetic supplier.

A spreadsheet was developed to calculate the total estimated costs associated with incorporating genome-enabled selection into a swine breeding program. The spreadsheet can calculate the accumulated costs for up to six different genetic lines that could be used to create a commercial animal. The nucleus herd size and nucleus production levels (i.e. number born alive, litters per sow per year, mortality, and boar production) can be changed to reflect the production system of the user. Which selection candidates are sampled on an annual basis can be altered to depict the strategy that the company anticipates implementing. Similarly, the spreadsheet can account for additional phenotyping costs associated with a novel trait being added to the selection criteria. Additionally, the spreadsheet can account for a multiplication factor if the genetic supplier has a multiplication level within their system.

## Results and discussion

The total start-up cost was US$326 031 for both maternal lines and US$318 338 for the terminal line. This includes the costs associated with developing the genome-enabled EBV and the genotyping of the initial dataset. The annual costs of routine genotyping were US$1 569 525 and US$361 758 for the maternal and terminal lines, respectively. Assuming a three-year planning horizon and a 5% discounting rate [4], the increased revenue for the maternal and terminal lines must be US$1 698 814.68 and US$491 047.68, respectively, in order to break even on the increased costs associated with incorporating genomic information into the selection program. Infrastructure costs are relatively small compared to genotyping costs; therefore, the annual total cost and cost per weaned pig are relatively insensitive to changes in the infrastructure costs.

Investments are routinely evaluated for their return on investment, which in turn is often annualized in order to evaluate yearly expenses relative to income. In the swine industry, genetic companies will incur the costs associated with genome-enabled selection. How genetic suppliers market their animals will govern how the costs are recovered. Some companies may sell boars or replacements while other companies may receive royalties based on the number of pigs weaned by their customers. For this study, it was assumed that the genetic company making the investment in genome-enabled selection receives royalties from weaned pigs at the commercial level.

Dividing the annualized costs by the number of semen doses per year, results in costs of US$3.68 and US$0.18 per dose in the maternal and terminal lines, respectively. To calculate the cost per nucleus boar, the annualized cost was divided by the number of boars used for commercial production (male selection candidates × percent used). The cost per nucleus boar was US$3052.13 and US$147.62 for the maternal and terminal lines, respectively. Using the total number of female pigs produced annually from the maternal nucleus herds (1 940 845/2), it was determined that genome-enabled selection costs would be approximately US$2.92 per nucleus daughter (F1 cross between two maternal lines). This number was calculated from dividing the annualized costs by the number of productive sows produced from the nucleus boars, accounting for the gilt drop out percent. Assuming that each sow produced 35 weaned pigs in her lifetime, there is a US$0.083 cost per weaned pig in the commercial production system for each maternal line (US$2.92/35). The cost per weaned pig in the commercial herd was determined to be US$0.047 for the terminal line, assuming that 10 544 584 weaned pigs are produced annually. This means that US$0.21 per weaned pig from boars produced in the nucleus would need to be added to the genetic merit for each market pig in order to break even on the additional cost associated with genome-enabled selection for all three lines. The marketing structure for the genetic supplier will determine how the additional costs associated with genome-enabled selection can or will be recovered from commercial sales.

The current rate of genetic gain in the nucleus will determine the proportional increase in rate of genetic gain needed to recover genome-enabled selection costs. According to the National Swine Registry, the current rate of annual genetic gain for their terminal line index is US$0.30, US$0.40, and US$0.30 per weaned pig for the Duroc, Landrace, and Large White populations, respectively [13]. The traits, days to market, weight gained from weaned to slaughter, and feed efficiency were included in the terminal sire index [13]. The rate of genetic gain for number of born alive is 0.08 and 0.07 pigs per year for Landrace and Large White lines, respectively. Thus, the total annual genetic improvement for both maternal lines would be US$0.432 and US$0.321 for the Landrace and Large White lines, respectively. This rate of genetic gain may be lower than gains expected from most swine genetic companies. For each maternal line, the total genetic improvement value is calculated by multiplying the terminal improvement by 1 plus the increase in number of born alive ($0.40 × 1.08 for Landrace, and $0.30 × 1.07 for Large White).

**Figure 1:**
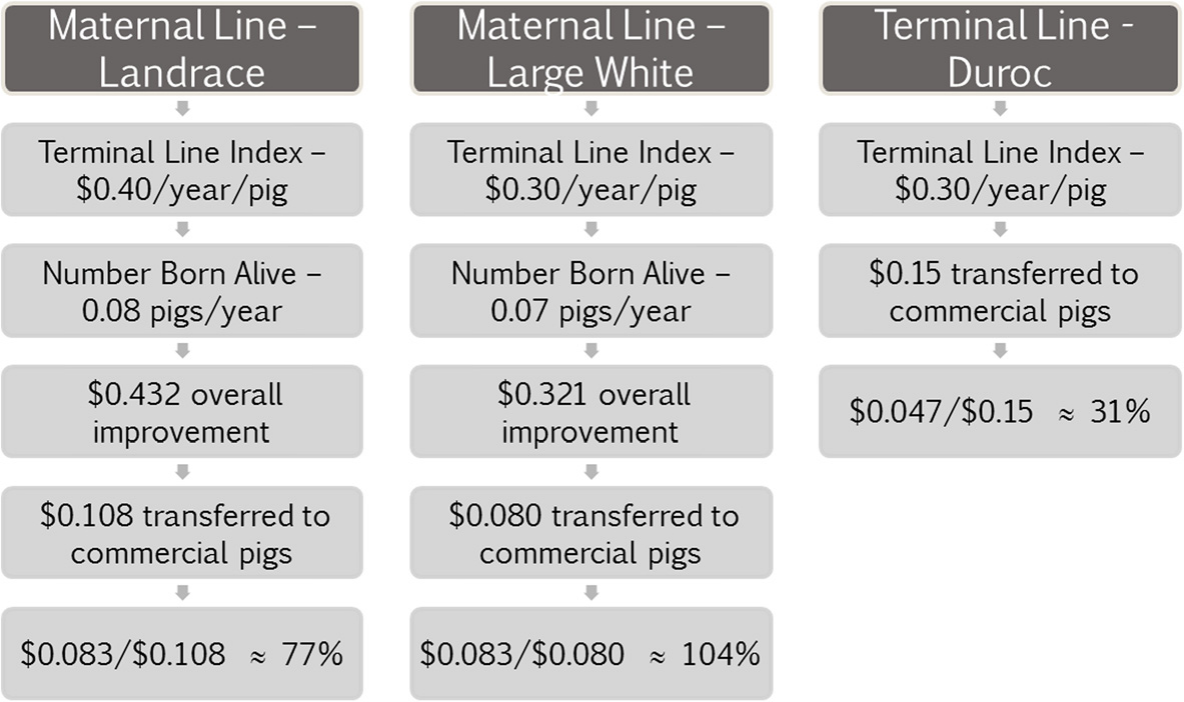
**Value of expected improvement in genetic merit at the commercial level.** Current rates of genetic improvement were based on genetic improvement published by the National Swine Registry for each of the three lines; the cost of genome-enabled selection was estimated using the values reported in this paper.

If the genetic supplier is part of an integrated system, then the total genetic improvement from a maternal line would be calculated as the terminal line improvement plus the carcass value of the additional pigs born alive. This would be $9.43 for Landrace and $8.20 for Large White assuming 25% mortality from birth to finisher, 114 kg market weight, and $1.32/kg market price. An integrated producer could recover the costs of genome-enabled selection by benefiting from improved production efficiency in the commercial segment or by increasing retail prices for the consumer while a genetic company would have to recover the costs of genome-enabled selection by increasing the premiums placed on their genetic product or by increasing market share. The increased price must be justified by an increased rate of genetic gain. A commercial producer will not necessarily pay for the full value of genetic improvement it will receive. The genetic supplier will only receive some percentage of the value of the improvement made based on the incremental improvement in genetic potential from one year to the next.The current expected improvement in genetic merit at the commercial level and the relative additional improvement needed to pay for incorporating genome-enabled selection into the breeding program are illustrated in Figure 1. Considering that 50% of the terminal line index improvement (US$0.15/weaned pig) in the Duroc line would be passed on to the commercial herd and that the cost estimate for genome-enabled selection in the terminal line was US$0.044 per weaned pig, this would mean that a 31% improvement (US$0.047/US$0.15) in the genetic gain rate would be needed to recover the genome-enabled selection costs. Considering this and the fact that 25% of the improvement occurring in each of the two maternal lines will be passed on to the commercial animals, 77% (US$0.083/US$0.108) and 104% (US$0.083/US$0.080) improvement in genetic gain rate is needed for the Landrace and Large White populations, respectively. Because of the planning horizon used in this study and by most swine genetic companies, these returns are based on one round of genetic improvement; however, differential genetic improvements made with genome-enabled selection will be accumulated over time.

These results reflect the necessary increase in genetic improvement at the nucleus level to break even on the estimated genome-enabled selection costs provided that the realized genetic improvement in commercial animals is equivalent to the expected genetic improvement. Environmental factors may reduce the realized genetic improvement at the commercial levels compared to the expected improvement based on predictions of genetic merit derived from data recorded in the nucleus production environment. Greater nucleus level genetic improvement would be required if all genetic improvement that occurs at the nucleus population is not realized or does not actually occur at the commercial production level.

**Figure 2:**
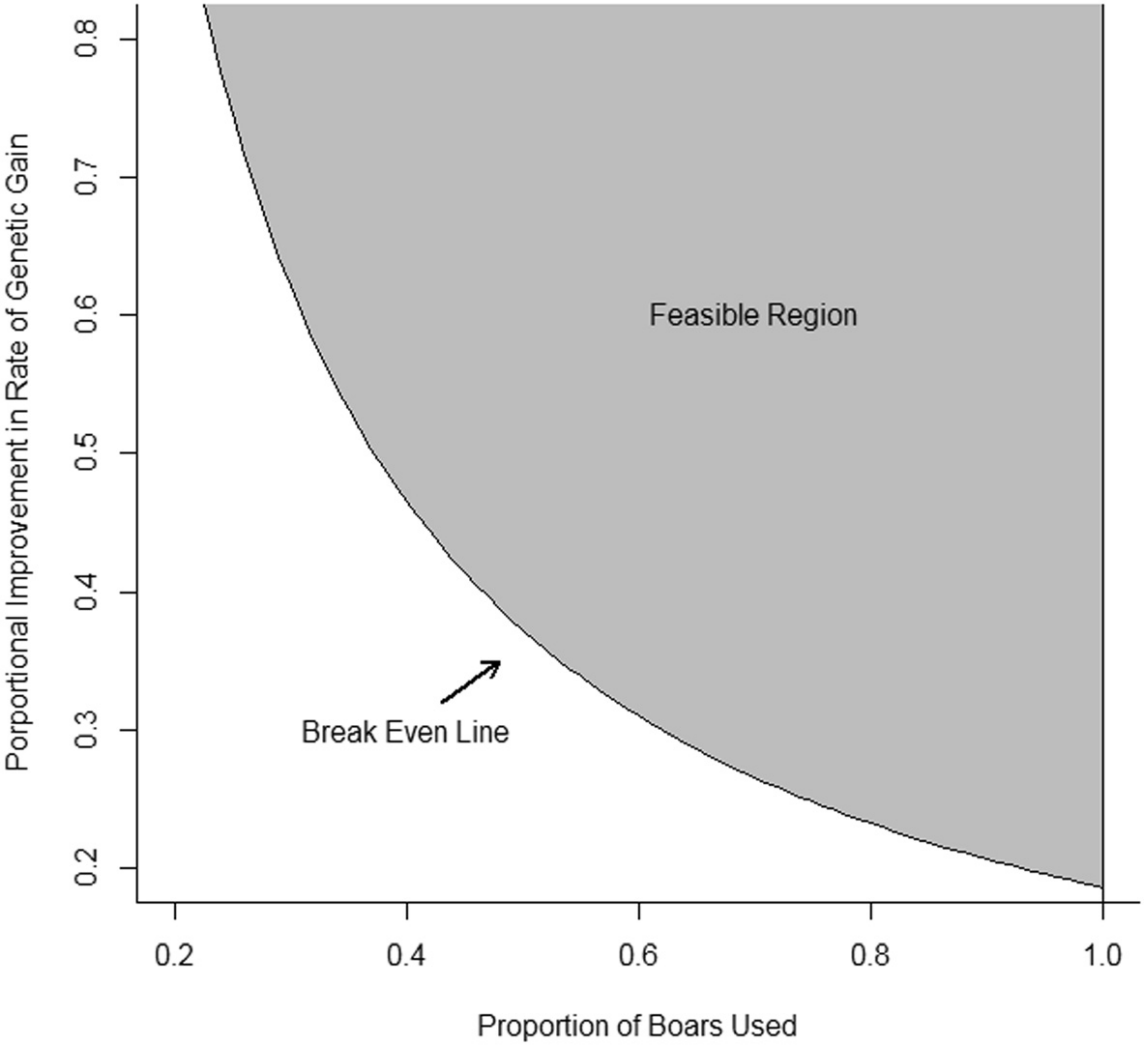
**Feasible region for profitability when incorporating genome-enabled selection in a terminal line selection program.** The current annual rate of genetic improvement was assumed to be US$0.15 per commercial pig; the number of commercial pigs produced from the nucleus boars was calculated using the values reported in the paper; the estimated cost of genome-enabled selection in the terminal line was US$491 048; this number was estimated using the strategy in this paper.

**Figure 3:**
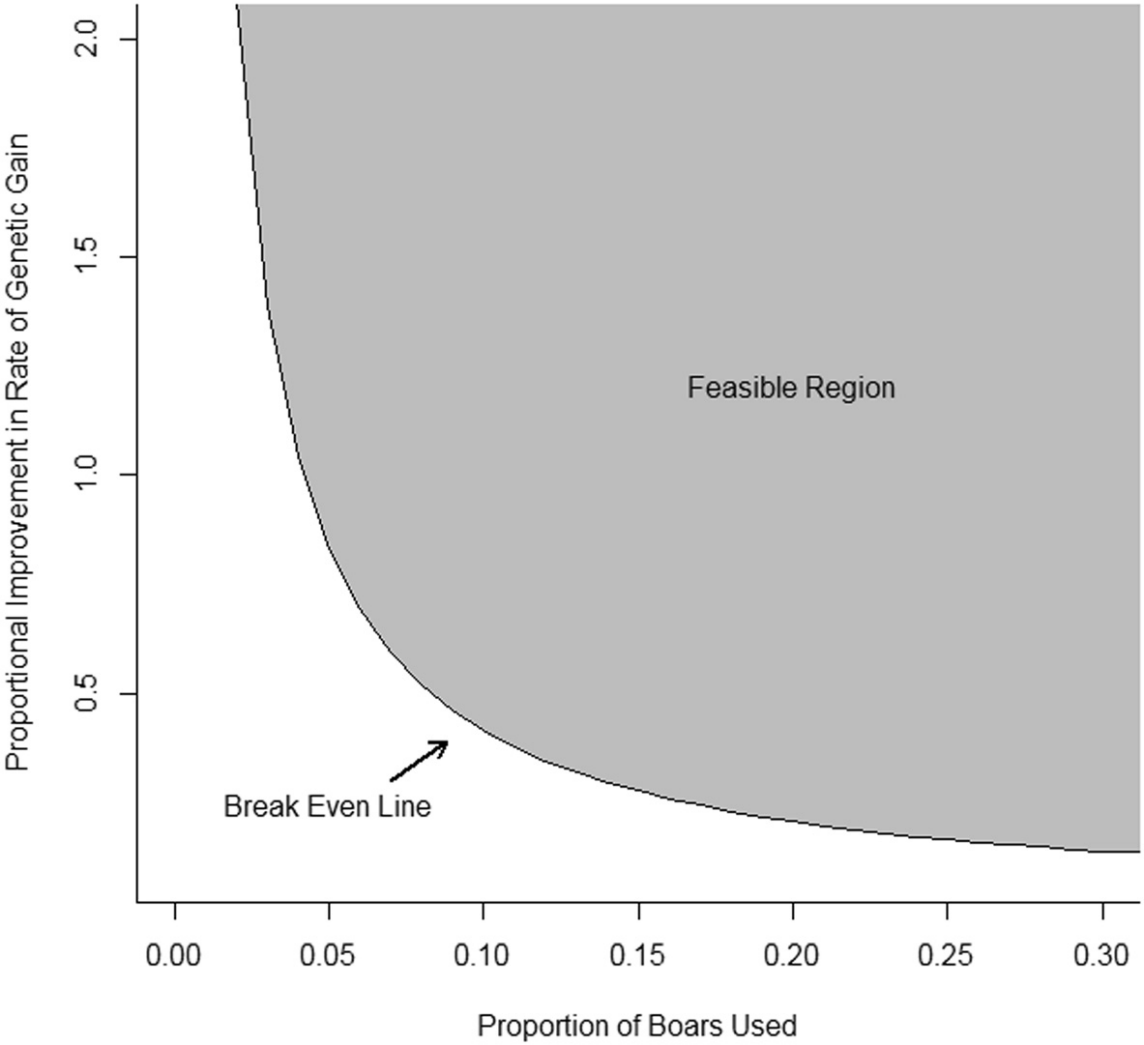
**Feasible region for profitability when incorporating genome-enabled selection in a maternal line selection program.** The current annual rate of genetic improvement was assumed to be US$0.10 per commercial pig; the number of commercial pigs produced from the nucleus boars was calculated using the values reported in the paper; the estimated cost of genome-enabled selection in the terminal line was US$1 698 815; this number was estimated using the strategy in this paper.

Another way to recover the costs associated with genome-enabled selection is through maintaining or capturing increased market share. If a company must invest in genome-enabled selection to maintain their current market share, there must be some perceived marketing value associated with incorporating genome-enabled selection into the breeding program. If a marketing value exists, the difference between the total costs associated with genome-enabled selection per weaned pig and the marketing value is the increased value in genetic improvement that must be achieved to break even on the investment in genome-enabled selection. Thus, the necessary increase in genetic improvement to break even would not be as large as if the costs must be recovered through increasing genetic potential alone.Without increasing the nucleus herd size, the only way to increase the market share is to increase the proportion of male offspring produced in the nucleus herd that are used to produce commercial piglets. The relationship between the proportion of nucleus boars used and proportion of improvement in genetic gain rate for the terminal and maternal lines are shown in Figures 2 and 3, respectively. For example, if 80% of the male candidates in the terminal line were used, compared to the 60% used in this study, the proportional improvement in rate of genetic gain needed to break even on the investment in genome-enabled selection would be 22% compared to 29%. The feasible region, or the region where the costs of genome-enabled selection are recovered, is shaded in gray. The gray area not on the break even line indicates that a profit is made. For the results in Figure 2, it was assumed that the current rate of genetic gain in the commercial animals was US$0.15 per pig from the terminal line. The current rate of genetic gain in the commercial animals was assumed to be US$0.10 per pig from the maternal line for the results in Figure 3.

The primary expected benefit from incorporating genomic information into EBV estimation is improved accuracy [2]. Due to the direct relationship between accuracy and rate of genetic gain, increasing EBV accuracy will proportionally increase the rate of genetic gain expected given that selection intensity and generation interval remain constant. This increase in accuracy will have to be sufficiently large to recover the added costs associated with using genomic information in the selection program.

Genome-enabled selection will not eliminate the need for phenotype collection. Earlier theories associated with genome-enabled selection suggested that one benefit would be the cost savings associated with reducing or eliminating the collection of phenotypic data [14]; however, due to the decay in accuracy associated with genomic breeding values over generations, genomic breeding values must be re-estimated periodically and phenotypic records will be needed [15].

The application of genomic breeding values has been investigated for pig populations. Nielsen and coworkers showed the correlation between genomic breeding values and traditional BLUP breeding values to be 0.62 for the 170 boars used in their data set [14]. Cleveland and collaborators reported the accuracy of the genomic breeding value for the total number of pigs born per litter to be between 0.64 and 0.82, depending on the initial dataset used [16]. The authors reported that the accuracy of stillborns per litter ranged from 0.33 to 0.68.

Dekkers [17] developed a method using selection index theory to calculate the genetic response expected from incorporating genomic information into a selection index. The method deterministically calculated the genetic response anticipated from using genomic selection with defined genetic parameters. The study showed that, for a trait recorded on both sexes prior to selection, selection based on markers alone can improve response by 8.5% compared to selection based only on phenotypic information. Based on stochastic simulations, annual genetic gain could be increased by 23 to 91% for a maternal line [5] and 27 to 33% for a terminal line [16]. Based on these increases in annual genetic gain, there is potential for genome-enabled selection to be profitable for both maternal and terminal line selection programs. Under simulated conditions, MAS could increase genetic improvement from selection for meat quality, net or residual feed intake, and number of pigs born alive compared to the response from traditional BLUP. Under these simulated conditions, the largest gap between marker-assisted selection and traditional BLUP occurred for the meat quality traits; however, no genetic improvement in rate of genetic gain was observed for growth when comparing marker-assisted and traditional BLUP selection methods under these simulated conditions [1]. This suggests that the greatest potential for improvement in the rate of genetic gain from genome-enabled selection will be for lowly heritable traits that are difficult to measure, sex limited, measured later in life or measured after slaughter, such as meat quality, disease resistance, feed efficiency, and longevity.

Disease resistance is not easily defined and not systematically measured. Feed efficiency is expensive to measure directly, especially on an individual animal basis. Sow longevity is not recorded until the sow is culled from the herd and is a trait that is only measured on females. If traits are not currently measured and recorded, additional costs associated with measuring the novel traits will be connected with genome-enabled selection if these traits are targeted in a selection program. For a novel trait to be incorporated into a selection program, a measureable phenotype associated with the trait must be clearly defined. Depending on the phenotype, there may be a significant cost associated with the infrastructure needed to collect the data.

## Conclusions

Using genomic information to estimate an animal’s genetic merit at the molecular level can improve EBV accuracy when compared to an EBV based only on phenotypic records. However, genome-enabled selection is expensive and the increase in rate of genetic gain must be large enough to offset the costs associated with incorporating genome-enabled selection into a breeding program. A flexible spreadsheet tool (accessible at the Iowa Pork Industry Center website: http://www.ipic.iastate.edu/software.html) developed from this work can be used to estimate the returns needed to recover additional costs associated with genome-enabled selection by modifying the input values such as herd size and genotyping strategy to represent the specific design of any production system.

## Competing interests

The authors declare that they have no competing interests.

## Authors’ contributions

JD, MR, and JM provided technical information and support for the project. CA and KS conceived the project, participated in spreadsheet development, and drafted the manuscript. All authors read and approved the final manuscript.
